# CAG repeat polymorphism in androgen receptor and infertility: A case-control study

**DOI:** 10.18502/ijrm.v19i9.9717

**Published:** 2021-10-10

**Authors:** Shiva Sharestani, Seyed Mehdi Kalantar, Nasrin Ghasemi, Ehsan Farashahi Yazd

**Affiliations:** ^1^Shahid Sadoughi University of Medical Sciences, Yazd, Iran.; ^2^Abortion Research Centre, Yazd Reproductive Sciences Institute, Shahid Sadoughi University of Medical Science, Yazd, Iran.; ^3^Department of Genetics, Shahid Sadoughi University of Medical Sciences, Yazd, Iran.; ^4^Stem Cell Biology Research Center, Yazd Reproductive Sciences Institute, Shahid Sadoughi University of Medical Sciences, Yazd, Iran.

**Keywords:** Infertility, Azoospermia, Androgens, X chromosome, Spermatogenesis.

## Abstract

**Background:**

Androgens play a role in the development of male phenotype and spermatogenesis during puberty, the function of which is regulated by the androgen receptor (*AR*) gene. There is a polymorphism site in exon 1 of the gene encoding this receptor that can have different frequencies of CAG trinucleotide repeats and leads to the formation of polyglutamine chains of different lengths in the N-terminal domain of the AR protein and reduced sperm production by affecting spermatogenesis.

**Objective:**

To investigate whether the cause of a group of unexplained infertilities could be the increased frequency of CAG repeats in the *AR* gene of patients with oligozoospermia and azoospermia.

**Materials and Methods:**

In this case-control study, 84 men including 42 with unexplained infertility As a case group and 42 fertile men as a control group were selected. The frequency of CAG repeats was determined by the polymerase chain reaction method and then the difference in the frequency of these repeats was determined based on the difference in band size on the agarose gel.

**Results:**

The mean CAG repeat length in the azoospermia and oligozoospermia group was 17.5 ± 0.63 and in the fertile group it was 16.11 ± 0.75 (p = 0.46). In addition, most men (88.1% in the case group and 71.41% in the control group) had 13-23 repeats.

**Conclusion:**

No significant correlation was found between CAG repeat length and the risk of male factor infertility in an ethnically defined population of Iranian men. The role of regulatory factors and epigenetic changes should be taken into account too.

## 1. Introduction

There is no standard definition for infertility. The World Health Organization defines infertility as a disease of the reproductive system defined by the failure to achieve a clinical pregnancy after 12 months or more of regular unprotected sexual intercourse (1). There are several factors that can contribute to infertility, depending on gender (2). Some infertility cases are caused by unexplained factors. Infertility is a genetically heterogeneous disease with a multifactorial etiology. Infertility is a big health problem worldwide as it has been estimated that in 2010 there were roughly 48.5 million infertile couples worldwide (3). Infertile men may develop phenotypic or sperm abnormalities (4). The development of male phenotype and spermatogenesis depend on cellular events that respond to androgens. Impaired spermatogenesis can be caused by a variety of factors, including genetic changes and mutations in genes involved in spermatogenesis, such as the gene encoding the androgen receptor (*AR*) (5). The action of androgens is mediated by the *AR*, which is encoded by a single copy gene located on the long arm of the X chromosome. This gene contains eight exons and encodes an intracellular transcription factor belonging to the steroid nuclear receptor superfamily. One of the polymorphisms that can be studied in this gene is a CAG trinucleotide repeat in exon 1 and the N-terminal domain of this gene, the product of which is polyglutamine. CAG repeats encode polyglutamine, and these strands enter the structure of the protein being synthesized. Mobasseri and colleagues studied a population of infertile and fertile Egyptian men using polymerase chain reaction and sequencing and found a significant relationship between increased CAG repeats in the *AR* gene and the development of oligozoospermia (OS) and azoospermia (AS) in infertile men (6). Some studies reported that this polymorphism may not be a significant factor in infertility in men (7). However some studies determined the frequency of CAG repeats in studied samples by PCR and sequencing and suggested the CAG polymorphism as a factor affecting spermatogenesis (8, 9).

This study aimed to evaluate and compare CAG trinucleotide repeat length in the *AR* gene in fertile and infertile men referred to the infertility treatment center in Yazd province, and examine the relationship between increased repeats and sperm count disorders including OS and AS.

## 2. Materials and Methods

This case-control study was conducted at Yazd infertility Treatment Center in 2014. Characteristics of the study population are summarized in Table I.

The inclusion criteria were 18-40 yr old men unexplained male infertility (case) and having a healthy child (control). With normal serum levels of the hormones prolactin, TSH, LH, FSH, and testosterone.

Men with obstructive azoospermia, male infertility due to Y chromosome micro deletions. Male infertility with an explained genetic cause such as cystic fibrosis, and abnormal level of hormones associated with fertility were excluded.

**Figure 1:**
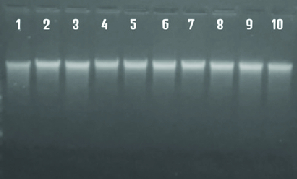
Agarose gel electrophoresis of DNA samples.

The sample size needed for each group was calculated as 42, based on confidence level of 95%, a minimum error of 5%, and the frequency of CAG trinucleotide repeats in case and control groups. After collecting all the samples, DNA was extracted using a Sinagene DNA Extraction Kit (Sinagene company, manufactured by Iran), 50 preps. A picture showing some of them is given below (Figure 1). PCR was performed to amplify DNA fragments of exon 1 of the *AR* gene. Then, the number of polyglutamine fragments was determined by electrophoresis on a polyacrylamide gel and staining with ethidium bromide (Sinagene Company, manufactured by Iran). CAG repeats of exon 1 of the *AR* gene were amplified by the following two primers.

Forward: 5'-GCT GTG AAG GTT GCT GTT CCT CAT-3'

Reverse: 5'-TCC AGA ATC TGT TCC AGA GCG TGC-3'

### Ethical considerations

The study protocol was approved by Shahid Sadoughi University of Medical Science Ethical Committee. Written informed consent was obtained from all participants before participating in the study.

### Statistical analysis 

PCR targeting followed by electrophoresis on a polyacrylamide gel were used for *AR*-CAG genotype detecting. A paired t test and Chi-square test was carried out to find the difference in CAG repeats between infertile and fertile men. Data were analyzed using the Statistical Package for the Social Sciences, version 20.0 (SPSS, USA). A p-value < 0.05 was considered significant.

## 3. Results

**Figure 2:**
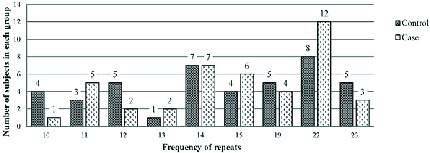
Comparison of allele frequencies in case and control groups.

A Chi-square test was used to investigate whether the distributions of the categorical variables differed from one another. The mean CAG repeat length in the As and Os group was 17.5 ± 0.63; and in the fertile with normal sperm count group, it was 16.11 ± 0.75. However, based on the results of the Chi-square test (p = 0.46), no significant correlation was found between the CAG repeat length and the risk of male factor infertility in the ethnically defined population of Iranian men, as shown in Figure 2 and Table I. In addition, most men (88.1% in the case group and 71.41% in the control group) had 13-23 repeats, and the lowest frequency in both case and control groups had 10-13 repeats (11.9% and 28.59%, respectively). The results of the calculations are shown in the diagram and table below (Table I, Figure 2).

**Table 1:** Characteristics of the control and case groups


**Group**	**CAG**	**p-value**	**Sperm count**	**Frequency**
**Control**	16.11 ± 0.75	Normal	42
<statement> <title>Case </title> </statement>	17.5 ± 0.63	0.46	OS–AS	42
Data presented as Mean ± SD. OS: Oligozoospermia, AS: Azoospemia, Paired *t* test and Chi-square test				

## 4. Discussion

Trinucleotide repeat expansion in the DNA of chromatin has been identified as the reason for ≥ 40 neurological disorders such as Fragile-X syndrome, Huntington's disease, inherited ataxias, and muscular dystrophy. Assuming that the genome architecture is important for genome function, evaluation of structural changes in chromatin may help to better understand mechanisms behind trinucleotide repeat expansion disease induction. Studies of patients and families with trinucleotide repeat disorders have revealed a number of genetic and molecular bases of trinucleotide repeat instability (5, 8, 9). Analysis of trinucleotide repeat instability in bacteria, yeast, and mice has yielded additional insights. Despite these advances, the pathways and mechanisms underlying trinucleotide repeat instability in humans remain largely unknown.

Investigations in vitro have demonstrated that the disease-causing repeats are capable of adopting non-B secondary structures that mediate repeat expansion. Although theoretically it may be easier for longer trinucleotide repeats to form non-B DNA secondary structures in replication or in post-replication, such non-B secondary structures are likely to cause repeat fragility rather than repeat expansion. In fact, repeat expansion may not necessarily require trinucleotide repeats to form non-B secondary structures; instead, the repeat expansions can be produced through a RNA transcription-stimulated local repeat DNA replication and a subsequent DNA rearrangement (10). The tendency of these trinucleotide repeats to expand is explained by the formation of alternative structures in DNA replication. Part of the array within the daughter strand can loop out without disrupting base-pairing outside this region. DNA polymerase extends this strand through the remainder of the array, leading to an increase in the number of copies of the trinucleotide sequence.

This study aimed to investigate whether the cause of a group of unexplained infertilities could be an increase in polyglutamine chain length due to an increased frequency of CAG repeats in the *AR* gene of patients with OS and AS; to this end, the relationship between CAG polymorphisms on exon 1 of the *AR* gene and disruption of the spermatogenesis pathway was examined. Some Studies on infertile men (11, 12) demonstrated that the size of the CAG repeat was higher in infertile men. Another studies on infertile men with defective spermatogenesis and idiopathic AS, respectively, demonstrated the presence of significantly longer CAG repeats compared with those in fertile men (8, 9). However, our observations were in agreement with the study (13) who examined 17 alleles in a population of infertile German men, ranging from 16 to 34 repeats, with the predominance of allele 21, whereas in the control group, the predominant allele was 23, which was slightly higher than that of infertile men. Recent studies have shown the importance of accurate data transfer as well as genetic and environmental factors for proper fertility (6, 14). Using this dataset, clinics will be better able to treat infertility and make better decisions about using assisted reproductive technologies. In some studies, a relationship between increased frequency of CAG repeats and OS and AS has been reported (6). However, a number of other clinical studies have found no significant relationship between the two groups (15-17). This clearly shows that what is true for one population may not be true for other populations and suggests that it would be detrimental to extrapolate such findings to other populations, particularly for diagnostic purposes.

##  Conflict of Interest 

The authors declare that there is no conflict of interest.
